# Clinical Presentation and Management of Methicillin-Resistant *Staphylococcus aureus* Pericarditis—Systematic Review

**DOI:** 10.3390/jcdd9040103

**Published:** 2022-03-30

**Authors:** Milan Radovanovic, Marija Petrovic, Richard D. Hanna, Charles W. Nordstrom, Andrew D. Calvin, Michel K. Barsoum, Natasa Milosavljevic, Djordje Jevtic, Mladen Sokanovic, Igor Dumic

**Affiliations:** 1 Mayo Clinic Alix School of Medicine, Rochester, MN 55905, USA; hanna.richard@mayo.edu (R.D.H.); nordstrom.cw@mayo.edu (C.W.N.); calvin.andrew@mayo.edu (A.D.C.); barsoum.michel@mayo.edu (M.K.B.); milosavljevic.natasa@mayo.edu (N.M.); dumic.igor@mayo.edu (I.D.); 2 Department of Hospital Medicine, Mayo Clinic Health System, Eau Claire, WI 54703, USA; 3 Icahn School of Medicine at Mount Sinai, New York, NY 10029, USA; petrovic_marija@yahoo.com (M.P.); djordje965@gmail.com (D.J.); 4 Department of Cardiology, Mayo Clinic Health System, Eau Claire, WI 54703, USA; 5 Department of Pediatric and Adolescent Medicine, Mayo Clinic Health System, Eau Claire, WI 54703, USA; 6 Department of Internal Medicine, Elmhurst Hospital Center, Elmhurst, NY 11373, USA; 7 Department of Infectious Disease, Jackson Hospital, Montgomery, AL 36106, USA; mladensokanovic@gmail.com

**Keywords:** methicillin-resistant *Staphylococcus aureus*, MRSA, purulent pericarditis, bacterial pericarditis

## Abstract

In the expanding era of antibiotic resistance, new strains of *Staphylococcus aureus* have emerged which possess resistance to traditionally used antibiotics (MRSA). Our review aimed to systematically synthesize information on previously described MRSA pericarditis cases. The only criterion for inclusion was the isolation of MRSA from the pericardial space. Our review included 30 adult and 9 pediatric patients (aged: 7 months to 78 years). Comorbid conditions were seen in most adult patients, whereas no comorbidities were noted amongst the pediatric patients. Pericardial effusion was found in 94.9% of cases, with evidence of tamponade in 83.8%. All cases isolated MRSA from pericardial fluid and 25 cases (64.1%) had positive blood cultures for MRSA. Pericardiocentesis and antibiotics were used in all patients. The mortality rate amongst adults was 20.5%, with a mean survival of 21.8 days, and attributed to multi-organ failure associated with septic shock. No mortality was observed in the pediatric population. In adult patients, there was no statistical difference in symptom duration, antibiotic duration, presence of tamponade, age, and sex in relation to survival. Conclusion: MRSA pericarditis often presents with sepsis and is associated with significant mortality. As such, a high clinical suspicion is needed to proceed with proper tests such as echocardiography and pericardiocentesis. In more than one third of the cases, MRSA pericarditis occurs even in the absence of documented bacteremia.

## 1. Introduction

Methicillin-resistant *Staphylococcus aureus* (MRSA) is a Gram-positive and coagulase-positive spherical pathogen that is a part of the *Staphylococcaceae* family. Infections due to MRSA are associated with higher mortality rates compared with methicillin-sensitive strains [1]. In healthcare settings, MRSA is a well-known nosocomial infection, accounting for at least 25 to 50% of *S. aureus* healthcare-associated infections (HA-MRSA) [2]. Outside of the healthcare setting, MRSA has emerged as one of the major causes of community-associated infections (CA-MRSA), causing skin and subcutaneous tissue infections in roughly 85% of cases [1,3]. Less frequently, it has been recognized as the cause of rapidly lethal and severe infections such as necrotizing pneumonia and fasciitis [1].

The major issue with MRSA infections is the remarkable level of resistance against multiple antibiotic classes, primarily due to the production of altered penicillin-binding protein (PBP) with decreased affinity for most semi-synthetic penicillins [1]. The genetic component behind altered PBP is located on the acquired *mecA* gene that is carried on a mobile genetic element (MGE)—designated staphylococcal cassette chromosome *mec* (SCC*mec*). To date, 13 SCC*mec* types have been identified [1,4]. CA-MRSA is genetically distinct from HA-MRSA, carrying a smaller version of SCC*mec* (types IV and V vs. types I–III present in HA-MRSA), and often producing the cytotoxin Panton–Valentine leukocidin (PVL) [1,5,6]. While larger SCC*mec* types I to III carry genes for resistance to multiple antibiotic classes (including non-β-lactam antibiotics), SCC*mec* types IV and V seen in CA-MRSA carry only the *mecA* gene for resistance to β-lactam antibiotics, which accounts for their non-multidrug-resistant phenotype [1]. Therefore, most CA-MRSA isolates are susceptible to fluoroquinolones, aminoglycosides, erythromycin, and clindamycin [7,8]. This, however, does not preclude resistance carried by plasmids which cause sporadic resistance to trimethoprim–sulfamethoxazole, clindamycin, tetracycline, vancomycin, gentamicin, fluoroquinolones, and macrolides [9].

MRSA causes the infection of numerous tissues and organ systems. When MRSA infection involves the heart, there is significant morbidity and mortality. The most common clinical presentation is endocarditis, while pericarditis and pericardial abscesses are sporadically reported. This study aims to describe clinical characteristics, diagnostic and therapeutic approaches, and the outcomes of patients suffering from microbiologically confirmed MRSA pericarditis in the published literature.

## 2. Materials and Methods

**Figure 1:**
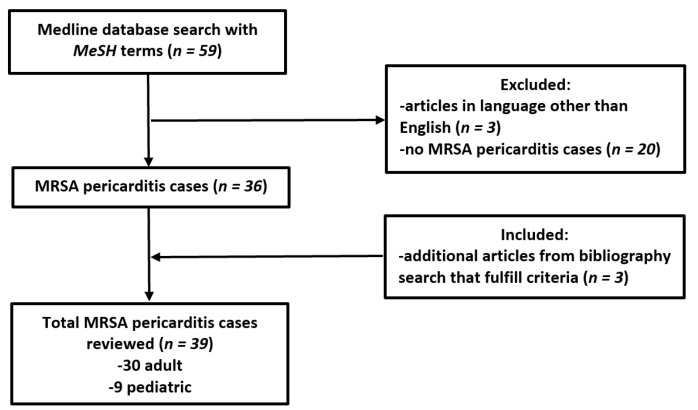
Flow chart of methodology and literature selection according to PRISMA guidelines.

We performed a systematic review of the literature according to Preferred Reporting Items for Systematic Reviews and Meta-Analyses (PRISMA) guidelines using PubMed/Medline (National Library of Medicine, Bethesda, MD) database, from database inception until 02/01/2022. A total of 56 original articles were found that mention MeSH terms: “Methicillin-resistant *Staphylococcus aureus* AND pericarditis” OR “MRSA AND pericarditis.” We excluded cases where the diagnosis was not certain either because pericardiocentesis was not performed or pericardial fluid was sterile. The flow chart of detailed article selection and the final cases included in the analysis is illustrated in Figure 1.

Two authors (M.R. and D.J.) independently and blindly identified and selected titles, abstracts, and full texts in the database search. Discrepancies of the selected articles were resolved by the senior author (I.D.). Subsequently, the reference list of selected articles was searched to identify any additional articles for inclusion in accordance with previously established selection criteria. An Excel table was constructed, and for each case we extracted patients’ demographic data, co-morbid conditions, presenting symptoms, laboratory and imaging findings (including electrocardiography (ECG), echocardiography, and computerized tomography (CT) scan), treatment options, complications, and outcomes.

Data were analyzed by descriptive statistics and expressed as mean ± standard deviation for continuous data, or as frequency and percentages for categorical data. The Student *t*-test and Chi-square tests were used to test the differences between patients in relation to outcome (survival). Statistical significance was reported using a *p*-value < 0.05. SPSS statistical software (version 21.0) was used for statistical analysis.

## 3. Results

### 3.1. Demographics and Comorbidities

Our systematic review identified 39 unique patients from 33 case reports describing a single patient, and three case series that described two patients each [10,11,12,13,14,15,16,17,18,19,20,21,22,23,24,25,26,27,28,29,30,31,32,33,34,35,36,37,38,39,40,41,42,43,44,45]. The age of patients ranged from 7 months to 78 years (mean 38.5 years), including nine (23.1%) from the pediatric population (Table 1). Both genders were almost equally represented. Comorbidities were seen in the majority (86.7%) of the adult patients but were not seen amongst the pediatric patients. The most common comorbidities were diabetes mellitus (DM), advanced chronic kidney disease (CKD), end-stage renal disease (ESRD), history of cancer, and immunosuppression. Only two patients had previous pericardial disease, constrictive pericarditis [33] and uremic pericarditis [13], each requiring a pericardial window. Only one patient had recent thoracic surgery–lung resection for lung cancer, complicated by pyothorax that was felt to be a potential cause of pericarditis; however, the patient was also immunosuppressed due to chemotherapy received for lung cancer [27].

### 3.2. Presentation Symptoms

Patients presented with a wide array of symptoms including chest pain (38.5%), tachycardia (82%), dyspnea (74.4%), fever (61.5%), and hypotension or shock (59%). The majority of the patients (~70%) did not have recognized pericardial disease or tamponade on presentation. Only 38.5% had specific exam findings suggestive of pericardial disease, such as pulsus paradoxus, muffled heart sound, and/or pericardial friction rub on physical examination. The duration of symptoms prior to admission to the hospital was reported in two-thirds of the patients, ranging from 1 to 18 days (mean 7.1 ± 5.1 days).

### 3.3. Evaluation

Nearly half of the patients (48.7%) had reported abnormal ECG findings, mainly ST-segment elevation with or without PR-segment depression (28.2%), followed by sinus tachycardia (20.5%). ECG findings indicative of pericardial diseases such as low-voltage QRS complexes and electrical alternans were present only in 17.9% of the cases. Nearly all cases (97.4%) underwent transthoracic echocardiography (TTE), and the majority (94.4%) had pericardial effusion (Table 2). Circumferential pericardial effusion was present in 75.7%, while in 18.9% of cases pericardial loculations or septations were seen. Localized pericardial fluid collection (abscess) was reported in three patients [13,34,45]. Additional diagnostic imaging performed included: CT of the chest in nearly two-thirds of the cases, cardiac magnetic resonance imaging (cMRI) in two cases [20,31], and transesophageal echocardiography (TEE) in two cases [25,36].

Consistent with our inclusion criteria, diagnostic pericardiocentesis was completed with the isolation of MRSA in all reported cases. MRSA bacteremia was documented in 64.1% of cases. CA-MRSA was identified in 19 cases (48.7%), and HA-MRSA was identified in two cases (5.2%). SCC*mec* and PVL genotyping were each performed in only four cases (10.3%), identifying three CA-MRSA and one HA-MRSA isolate. Reporting of pericardial fluid analysis (cytology and biochemical analysis) was inconsistent and performed in approximately 30% of cases. Similarly, reporting of MRSA susceptibility was performed in only 38% of cases.

### 3.4. Treatment and Interventions

A combination of pericardial decompression with pericardiocentesis and antibiotic management was utilized in all patients (100%). In addition to pericardiocentesis, a pericardial drain was placed in 17 patients (43.6%) with a pericardial washout performed in seven patients (17.9%). Pericardiotomy and window were completed in 11 cases (28.2%) and pericardiectomy was performed in only four cases (10.3%). Fibrinolytic therapy was not used in any of our reviewed patients, although intrapericardial instillation of physiologic saline was used as an adjunct in the setting of loculated fibrinous collection [27].

The empiric antimicrobial treatment reported within the first 48 h of the presentation was documented in 59% of patients. After MRSA isolation, 33% received intravenous (IV) vancomycin alone, 29% received vancomycin in combination with other antibiotics (e.g., meropenem, linezolid, rifampin, gentamicin, or daptomycin), 23% received alternative antibiotics (e.g., clindamycin, linezolid, daptomycin, or ceftaroline), and 15% reported antibiotic use without mention of a specific regimen. The duration of the antibiotic course ranged from 4 days to 24 weeks (mean 6.5 ± 4.7 weeks).

Anti-inflammatory management, including non-steroidal anti-inflammatory medications (NSAIDs), colchicine, and steroids, was seldom used and was only reported in six cases (15.4%) (Table 3).

### 3.5. Complications and Outcome

The most common complications were septic shock with multiorgan failure (35.9%), followed by pleural effusions/empyemas and the re-accumulation of pericardial fluid (each 30.8%), and septic emboli (15.4%). Less commonly reported complications included pancarditis (7.9%), endocarditis (5.1%), and myocarditis (2.6%). Despite being reported in the literature as one of the most common complications of bacterial pericarditis [46,47], constrictive pericarditis was reported in only one patient [20].

The majority of patients had a positive outcome and recovered from infection (79.5%). Eight patients expired due to septic shock and multiorgan failure, with death occurring after a mean of 21.8 ± 15.3 days (range: 4 to 42 days) (Table 4). Out of those, seven had cardiac tamponade, and six had reported bacteremia. No mortality was observed in the pediatric population. There was no statistical difference in symptom duration, antibiotic duration, sex, and age in relation to outcome (survival) amongst adult patients. There was no difference between adult patients presenting with pulsus paradoxus, hypotension, and ECG findings with respect to survival or the presence of tamponade and its management (*p* > 0.05).

## 4. Discussion

In the pre-antibiotic era, *Streptococcus pneumoniae* was the most common cause of purulent pericarditis, with pneumonia being the most common primary source [46,48]. In the post-antibiotic, however, *S. aureus* has dominated [49]. It was not until 1991 that the first MRSA pericarditis case was reported [42], with a notable increase in cases in the past two decades, and a shifting epidemiology from HA-MRSA to CA-MRSA. The epidemiological and molecular distinctions between these two types of strains have become less defined, as numerous reports of CA-MRSA causing nosocomial outbreaks have been noted [1]. Current clinical practice does not embrace routinely performing molecular distinction between these two types, as management is not influenced by this information. Only four of our reviewed 39 cases had reported SCC*mec* and/or PVL genotyping, and this was pursued primarily for research purposes.

### 4.1. Risk Factors and Infection Mechanisms

Risk factors for purulent pericarditis are associated with preexisting pericardial injury (such as thoracic surgery, malignancy, penetrating trauma with pericardial contamination) or systemic processes including uremia, connective-tissue disease, or immunosuppression [46,48]. Our pediatric population did not have any preexisting systemic or known pericardial problems. Most of the cases (78%) were tested for primary immunodeficiencies that would predispose them to bacterial infections. One of the most important tests in the pediatric population is the interleukin-1 receptor-associated kinase 4 deficiency (IRAK-4), which predisposes children to severe and recurrent staphylococcal and pneumococcal infections [16,50,51]. Although no innate immunodeficiencies were discovered, one child received steroids for poison ivy one week before the presentation of MRSA pericarditis [31]. While influenza in children affects a variety of host defense mechanisms predisposing them to staphylococcal co-infection, leading to severe and fatal complications in previously healthy children [52], purulent pericarditis is a very rare complication of MRSA co-infection with influenza [53]. In the adult population with comorbidities, immunosuppression was observed in up to 70% with the most encountered traditional risk factors being uncontrolled DM in 23.1% and advanced CKD (including ESRD) in 19.2%. These conditions were, respectively, presumed to predispose patients to bone and soft tissue MRSA infections that later disseminated to the pericardium, or causing uremia and subsequent pericardial injury. Patients without comorbidities or traditional risk factors had the source of MRSA infection identified, which consisted of lower extremity cellulitis [41], elbow abscess [20], and thrombophlebitis of the internal jugular vein (Lemierre’s syndrome) [26].

Purulent pericarditis commonly occurs via one of the two mechanisms: hematogenous spread of the pathogen from a distant source, or direct spread from the surrounding structures to the pericardium (as in cases of pneumonia, pleural empyema, or subdiaphragmatic infection) [46]. In cases of endocarditis, the pericardial infection can occur due to hematogenous dissemination, but cases of myocardial destruction with periannular abscess and fistula formation to pericardium have been reported in the literature [54,55]. None of our patients had suspected direct spread of infection from the endocardium through the myocardium, but hematogenous spread (bacteremia) and direct spread from empyema were common (although it is difficult to establish exact causality as patients often are co-diagnosed with pericarditis, pleural empyema, and septic emboli on presentation). All three pancarditis cases [24,25,31] hypothesized hematogenous spread and septic emboli being the cause of pericarditis among other complications.

Unusual cases of direct spread of infection to pericardium have been reported, such as in the case of esophago-pericardial fistula caused by esophageal cancer [18] or internal jugular vein thrombophlebitis [26]. Patients with thoracic cancers treated with radiation therapy are at a higher risk of developing pericardial disease, which further increases the risk of pericarditis. This was observed in two patients suffering from breast [15] and lung cancer [27]. Unusual cases of a hematogenous spread from a distant source are also possible, for example in a case of MRSA endogenous endophthalmitis reported by Sheridan et al. [35]. Notably, there were two cases [38,43] that developed MRSA bacteremia and subsequent MRSA pericarditis following a percutaneous coronary intervention (PCI) in patients requiring coronary stents. Coronary artery instrumentation can result in iatrogenic bacteremia, with the incidence being around 1% [56], but various articles report incidence up to 7.3% immediately after catheterization [57].

### 4.2. Presentation and Diagnostics

The initial presentation of patients with MRSA pericarditis can be dramatic, but also non-specific, making further evaluation challenging, particularly when septic shock is the predominant feature. Expedited echocardiographic imaging, including point-of-care ultrasound (POCUS), can be valuable in establishing the diagnosis of pericardial effusion, and even in directing further investigations and treatment. Conversely, in patients with septic shock, POCUS-identified pericardial effusions may be mistaken for tamponade, leading to inappropriate and invasive management with pericardiocentesis [58]. There are other challenges, such as the potential absence of typical tamponade signs in the cases of severe pulmonary hypertension or severe right ventricular hypertrophy, as well as attenuated respiratory septal motion in the setting of the rigid hypertrophied septum [59]. Formal TTE is necessary for more precise diagnostic evaluation and further guidance [46,60]. In cases of non-diagnostic TTE with high suspicion of a pericardial effusion, additional cardiac (i.e., TEE, CT or cMRI) imaging could be invaluable to establish the diagnosis and assess complications. Contrast-enhanced CT of the chest can serve as an adjunct to evaluate pericardium, myocardium, and adjacent structures, and evaluate for complications, such as septic pulmonary emboli or septic aortic aneurysms [46]. In this review, we found that a CT scan was performed in 67% of cases, revealing pericardial effusion, pleural effusion(s), septic emboli, aortic pseudoaneurysm [41], or saccular (mycotic) aneurysm of the aortic arch [37]. In a case of Lemierre’s syndrome, thrombosis of the internal and external jugular, subclavian, and axillary veins [26]. TTE demonstrated tamponade in 83.8% of the cases. This finding highlights the severity of MRSA pericarditis, especially when compared with previous reports of tamponade in 14% of cases with idiopathic pericarditis and 61% of cases with neoplastic or purulent pericarditis [61]. 

Once a pericardial effusion is diagnosed in patients with a septic presentation, pericardiocentesis can be helpful in establishing the cause. Pericardial fluid in bacterial pericarditis may be frankly purulent, but additional laboratory data are necessary to further differentiate the nature of the effusion (i.e., cell count, glucose effusion to serum ratio, and lactate-dehydrogenase level and culture). Cultures should also be sent for bacterial, tuberculosis, and fungal studies [46]. Interestingly, more than one-third of patients with MRSA pericarditis did not have positive blood cultures. This might be explained either by blood cultures being falsely negative (various collection and incubation techniques in the cases were reported), or pericarditis occurring from a direct spread from surrounding structures with no hematogenous dissemination.

### 4.3. Treatment and Interventions

Vancomycin historically has been the drug of choice and sometimes the last resort for the treatment of serious MRSA infections, providing both initial empiric coverage and definitive therapy. Unfortunately, its increased use has diminished its effectiveness as an anti-staphylococcal agent, particularly with the development of vancomycin-intermediate *S. aureus* (VISA) and vancomycin-resistant *S. aureus* (VRSA) strains [62,63]. In our review, vancomycin alone or in combination with other anti-staphylococcal antibiotics was used in 62% of cases with overall good outcomes. The general recommendation for a fully immunized child with purulent pericarditis is an empiric treatment regimen that includes agents effective against MRSA, especially in communities where it is prevalent [17]. 

Recurrence of the effusion is common (30% reported in the literature, and more likely if heavily loculated) [46,48]. In our review, the reaccumulation of pericardial effusion was observed in 30.8% of cases. In such cases, the placement of a pericardial drain, pericardial window, or pericardiotomy is recommended (pericardial window being a Class I recommendation and subxiphoid pericardiotomy as a Class IIa based on the 2015 ESC Pericardial disease Taskforce guidelines) [46]. The role of the pericardial instillation of physiologic saline to prevent constriction and avoid pericardiectomy in patients with purulent pericarditis was successfully reported by Terada et al. [27]. This intervention was felt to be beneficial due to the high viscosity of purulent effusion with fibrinous debris and the inability of complete drainage by pericardiocentesis. Additionally, daily intrapericardial washouts were found to decrease the inflammatory reaction, thereby preventing constrictive pericarditis as a late complication. Further adjunctive therapy with intrapericardial fibrinolytic infusion has been recommended for loculated effusions to accelerate effective drainage (Class IIa per 2015 ESC Pericardial disease Taskforce guidelines) [46]. Fibrinolytic therapy was felt to offer a promising alternative to invasive surgical interventions, although current data on efficacy are mostly limited to case reports and case series [28,64]. Potential complications such as left ventricular and submitral pseudoaneurysms due to the focal accumulation of thrombolytic during the treatment have been reported [65,66]. Ineffective drainage in cases of loculations and fibrinous formations often requires a pericardial window, pericardiotomy, or extensive pericardiectomy to achieve adequate drainage [46,67].

### 4.4. Complications and Outcome

Although all patients in our review received drainage and antibiotics, we found that mortality was 20.5% lower than traditionally reported in the literature for bacterial pericarditis (40%) [47]. This might be explained by publication bias as authors are more likely to report good outcomes in such a life-threatening condition, and all our reviewed cases had pericardial drainage, as our selection criteria was MRSA isolation from pericardial space, which should yield a better prognosis. In comparison, MRSA endocarditis carries a mortality of 40–80% and is even higher in patients with prosthetic valves [68,69]. Comparatively, mortality for patients with fungal endocarditis is above 80% [56].

Constrictive pericarditis has been reported to occur rarely (<3%) in patients with purulent (non-tuberculous) pericarditis [46,48,70]; our review is generally consistent with this, although it may be under-reported due to the lack of long-term follow-up in the majority of case reports and case series.

## 5. Conclusions

MRSA pericarditis is potentially a fatal condition that should be included in the differential diagnosis of patients presenting with shock. Making the diagnosis can be challenging since most cases present with non-specific signs and symptoms of infection. Moreover, bacteremia was absent in over one-third of patients. As such, an appropriate level of clinical suspicion is needed. Additionally, we advise POCUS or TTE to be used early in the assessment of hemodynamically unstable patients, thus facilitating early diagnosis of pericardial effusion and tamponade. With prompt treatment with a combination of pericardial drainage and systemic antibiotics, the risk of mortality with MRSA pericarditis can be minimized.

## 6. Highlights

The published case studies indicate that:

- MRSA pericarditis often presents with sepsis and is associated with significant mortality.
- In more than one-third of cases, MRSA pericarditis occurs even in the absence of documented bacteria.
- MRSA pericarditis can occur in children and patients without serious underlying co-morbidities.
- Pericardial effusion may be seen incidentally on chest or abdominal CT imaging.
- POCUS is a promising tool to assist in rapidly guiding further investigations.
- Pericardiocentesis is necessary whenever the purulent pericarditis diagnosis is suspected.
- Reaccumulation of fluid after pericardiocentesis was common, arguing for continuous drainage after pericardiocentesis.
- MRSA pericarditis is more likely to lead to pericardial tamponade (83.8%) than idiopathic (14%) or neoplastic (61%) pericarditis.
- Despite antibiotic use and pericardial drainage, mortality remains high (20.5%, mean survival of 21.8 days) due to multi-organ failure associated with septic shock.

## Figures and Tables

**Table 1:** Patients’ demographics in MRSA pericarditis cases.

Demographic Characteristics	n	M to F Ratio	Age Range (Years)	Mean Age (Years)
Adult	30 (76.9%)	8:7	18–78	48.4 ± 16
Pediatric	9 (23.1%)	4:5	0.6–15	5.6 ± 4.7
Total	39 (100%)	20:19	0.6–78	38.5 ± 23
** Co-morbidities in the adult population**				
Present	26 (86.7%)			
Immunosuppression	18 (69.2%)			
Diabetes Mellitus	6 (23.1%)			
Advanced CKD/ESRD	5 (19.2%)			
Active cancer	3 (11.5%)			
HIV/AIDS	2 (7.7%)			
Liver transplant	1 (3.8%)			
Splenectomy	1 (3.8%)			
Recent chest surgery/PCI	6 (23.1%)			
Coronary artery disease	3 (11.5%)			
Chronic hepatitis B or C	3 (11.5%)			
Smoking/alcoholism/drug abuse	3 (11.5%)			
History of cancer in remission	2 (7.7%)			
Pericarditis or previous pericardial window	2 (7.7%)			
Not present	4 (13.3%)			

Legend: M—male; F—female; PCI—percutaneous coronary intervention; CKD—chronic kidney disease; ESRD—end-stage renal disease; HIV—human immunodeficiency virus; AIDS—acquired immunodeficiency syndrome.

**Table 2:** Diagnostic findings in MRSA pericarditis cases.

**ECG Findings**	
Normal or not reported	20 (51.3%)
Abnormal	19 (48.7%)
ST-elevation and/or PR-depression	11 (28.2%)
Sinus Tachycardia	8 (20.5%)
Low voltage QRS complexes	6 (15.4%)
Atrial fibrillation	3 (7.7%)
Electrical alternans	1 (2.6%)
**Echocardiography findings**	
Pericardial effusion	37 (94.9%)
With tamponade physiology	31 (83.8%)
Without tamponade physiology	6 (16.2%)
Circumferential effusion	28 (75.7%)
Effusion with loculations/septations	7 (18.9%)
Pericardial abscess	2 (5.4%)
Constrictive pericarditis with abscess	1 (2.6%)
Not reported (pericardial abscess seen on chest CT scan)	1 (2.6%)

**Table 3:** Treatment and outcome in MRSA pericarditis cases.

Treatment	
Antibiotics	39 (100%)
Pericardiocentesis	39 (100%)
Pericardial drain	17 (43.6%)
Pericardial window/Pericardiotomy	11 (28.2%)
Pericardial washout	7 (17.9%)
Pericardiectomy	4 (10.3%)
Anti-inflammatory therapy	6 (15.4%)
Fibrinolytic therapy	0 (0%)
**Complications and outcome**	
Recovered	31 (79.5%)
Pleural effusion/empyema	12 (30.8%)
Re-accumulation of pericardial effusion	12 (30.8%)
Septic shock	6 (15.4%)
Septic emboli	6 (15.4%)
Constrictive pericarditis	1 (2.6%)
Death	8 (20.5%)
Septic shock/multi-organ failure	8 (20.5%)

**Table 4:** Published cases reporting fatal MRSA pericarditis.

Reference	Age/Sex	Comorbidities	Symptom Duration	Bacteremia	Pericardial Finding	Pericardial Drainage	MRSA Type	Genotyping	Time to Death
Tan TL et al (2020) [11]	44 M	DMT2, foot osteomyelitis, chronic HBV infection	2 days	Yes	Tamponade	Pericardiocentesis (300 mL)	CA-MRSA	-	42 days
Kariyanna et al. (2018) [18]	54 F	Esophageal cancer, esophageo-pericardial fistula	2 weeks	No	Tamponade	Pericardiocentesis	CA-MRSA	-	not reported
Shihadeh et al. (2017) [22]	29 F	DMT2, recent hair transplant and scalp abscesses	2 days	Yes	Tamponade	Pericardiectomy	CA-MRSA	PVL gene	4 days
Kumar et al. (2013) [29]	78 M	HTN, CKD 4, SCC of buccal mucosa	10 days	Yes	Tamponade	Pericardiocentesis (800 mL)	MRSA	-	4 days
Hara et al. (2013) [33]	67 M	Constrictive pericarditis, liver cirrhosis due to HCV	-	Yes	Constrictive pericarditis	Diagnosed on autopsy	MRSA	-	31 days
Sheridan et al. (2010) [35]	53 F	CKD, CAD, Endogenous endophthalmitis	-	Yes	Tamponade	Pericardiocentesis	CA-MRSA	SCC*mec* IV	4 weeks
Saito et al. (2009) [37]	66 F	Not reported	2 weeks	Yes	Tamponade	Pericardial window (500 mL)	MRSA	-	not reported
Durao et al. (2008) [40]	31 F	Liver transplant	-	-	Tamponade	Pericardiocentesis (1000 mL)	MRSA	-	not reported

Legend: DMT2—diabetes mellitus type 2; HBV—Hepatitis B virus; HTN—hypertension; CKD—chronic kidney disease; HCV—Hepatitis C virus; CAD—coronary artery disease; CA-MRSA—community-acquired MRSA; SCC*mec*—staphylococcal chromosomal cassette; PVL—Panton–Valentine leucocidin.
